# Reperfusion strategies in stroke due to isolated cervical internal carotid artery occlusion: systematic review and treatment comparison

**DOI:** 10.1007/s10072-020-04735-5

**Published:** 2020-10-10

**Authors:** Michele Romoli, Maria Giulia Mosconi, Patrizia Pierini, Andrea Alberti, Michele Venti, Valeria Caso, Simone Vidale, Enrico Maria Lotti, Marco Longoni, Paolo Calabresi, Georgios Tsivgoulis, Maurizio Paciaroni

**Affiliations:** 1 Neurology Clinic, University of Perugia–S. Maria della Misericordia Hospital, Perugia, Italy; 2 Neurology Unit, Rimini “Infermi” Hospital–AUSL Romagna, Viale Settembrini 2, 47923 Rimini, Italy; 3 Stroke Unit and Division of Cardiovascular Medicine, University of Perugia–S. Maria della Misericordia Hospital, Perugia, Italy; 4 Neurology Unit, “M. Bufalini” Hospital–AUSL Romagna, Cesena, Italy; 5 Institute of Neurology, Catholic University of Sacred Heart, Roma, Italy; 6 Fondazione Policlinico Universitario A. Gemelli IRCCS, Roma, Italy; 7 Second Department of Neurology, “Attikon” Hospital School of Medicine, National and Kapodistrian University of Athens, Athens, Greece; 8 Department of Neurology, University of Tennessee Health Science Center, Memphis, TN USA

**Keywords:** Thrombectomy, Thrombolysis, stroke, Ischemic stroke, Internal carotid artery

## Abstract

**Introduction:**

Despite intravenous thrombolysis (IVT) and endovascular treatment (EVT) have been demonstrated effective in acute ischemic stroke (AIS) due to large vessel occlusions, there are still no conclusive data to guide treatment in stroke due to cervical internal carotid artery (ICA) occlusion. We systematically reviewed available literature to compare IVT, EVT, and bridging (IVT + EVT) and define optimal treatment.

**Methods:**

Systematic review followed predefined protocol (Open-Science-Framework osf.io/bfykj). MEDLINE, EMBASE, and Cochrane CENTRAL were searched. Results were restricted to studies in English, with sample size ≥ 10 and follow-up ≥30 days. Primary outcomes were favorable outcome (mRS ≤ 2), mortality, and symptomatic intracerebral hemorrhage(sICH), defined according to study original report. Newcastle-Ottawa scale was used for bias assessment.

**Results:**

Seven records of 930 screened were included in meta-analysis. Quality of studies was low-to-fair in 5, good in 2. IVT (*n* = 450) did not differ for favorable outcome and mortality compared to EVT (*n* = 150), though having lower rate of sICH (OR = 0.4, 95% CI 0.2–0.8). Compared to IVT, bridging (IVT + EVT) was associated with higher rate of favorable outcome (OR = 2.2, 95% CI 1.3–3.7). Compared to EVT, bridging (IVT + EVT) provided higher rate of favorable outcome (OR = 1.9, 95% CI 1.1–3.4), with a marginally increased risk of sICH (OR = 2.1, 95% CI 1–4.4) but similar mortality rates.

**Conclusions:**

Our systematic review highlights that, in acute ischemic stroke associated with isolated cervical ICA occlusion, bridging (IVT + EVT) might lead to higher rate of functional independence at follow-up, without increasing mortality. The low quality of available studies prevents from drawing firm conclusions, and randomized-controlled clinical trials are critically needed to define optimal treatment in this AIS subgroup.

**Electronic supplementary material:**

The online version of this article (10.1007/s10072-020-04735-5) contains supplementary material, which is available to authorized users.

## Introduction

Intravenous thrombolysis (IVT) with recombinant tissue plasminogen activator (rtPA) is approved as a first-line treatment for acute ischemic stroke within appropriate time-frame [1]. In recent years, a bulk of literature has accumulated supporting the effectiveness and safety of endovascular treatment (EVT), which has therefore been implemented in current guidelines as bridging (IVT + EVT) or as direct intervention [1].

Previous randomized-controlled clinical trials (RCTs) and observational studies have highlighted differences in the effectiveness of reperfusion strategies depending on occlusion site [2–4]. In particular, strokes due to acute internal carotid artery (ICA) occlusion are associated with poor prognosis [3, 4]. Occlusion of extracranial ICA has very low recanalization rates, so that poor benefit from IVT alone has been postulated [3, 5]. A previous systematic review highlighted that EVT might provide higher rates of favorable outcome, though associating with significantly higher risk of symptomatic intracerebral hemorrhage (sICH) [4]. However, results were derived from studies with interventions mostly limited to what nowadays is considered a restricted time-window. Moreover, data from studies using IVT before EVT were pooled in EVT group, and ICA site of occlusion varied from cervical to terminal or tandem [4], preventing from drawing meaningful conclusions on the optimal reperfusion strategy.

To date, no completed RCTs exist addressing the specific question of the optimal reperfusion treatment in stroke due to extracranial ICA occlusion. Moreover, despite a vast amount of literature produced on stroke due to large vessel occlusions [6], there are still no conclusive clinical data to guide best treatment approach in extracranial ICA occlusion-related stroke.

Here we provide a systematic analysis of published studies to define clinical outcomes in patients stroke due to occlusion extracranial ICA treated with IVT, EVT, or bridging therapy.

## Methods

### Search strategy and selection criteria of the systematic review

Systematic review followed PRISMA guidelines and protocol registered with Open Science Framework (osf.io/bfykj). MEDLINE, EMBASE, and Cochrane Central Register for Controlled Trials (CENTRAL) were searched up to March 04, 2020 for studies reporting on treatment of acute ischemic stroke due to occlusion of extracranial ICA. Search string included combination of the following terms: (i) “stroke” OR “cerebrovascular diseas*”; (ii) “tissue plasminogen activator” OR “thromboly*” OR “rtPA” OR “alteplase” OR “tenecteplase”; (iii) “endovascular procedure*” OR “endovascular treatment” OR “thrombectomy” OR “aspiration” OR “retrieval”; (iv) “internal carotid artery” OR “proximal carotid” OR “extracranial carotid” OR “cervical carotid” (“*” as catch-term). We restricted results to studies with isolated extracranial ICA occlusion documented by ultrasound (US), computed tomographic angiography (CTA), magnetic resonance angiography (MRA) or digital subtraction angiography (DSA), and with sample size of treated patients ≥ 10 and follow-up of at least 30 days in order to minimize anecdotal report-related bias [4]. Only studies in English were included. Two researchers independently carried out the search and identified eligible studies. Controversies were resolved by the senior author.

### Data extraction, bias assessment, and defined outcomes

Two authors extracted data from eligible papers, including design, setting, sample size, reperfusion strategy (IVT, EVT, or IVT + EVT). For studies published more than once (i.e., duplicates) and with multiple time-points, we included only the report with the most informative and complete data. Outcomes were (i) favorable outcome, according to study original definition, (ii) mortality, and (iii) sICH. For included studies, successful reperfusion according to in-study definition was extracted as secondary outcome. When specific definition of sICH was not available, we considered as sICH (i) hemorrhages associated with decline in neurological status and (ii) parenchymal hematomas. Studies were excluded from respective analysis if they did not provide data on clinical outcomes predefined. Studies reporting outcomes at < 30 days were excluded. Three groups of reperfusion strategies were defined (IVT, EVT, or IVT + EVT), with clinical outcomes extracted and attributed according to study reports. Cochrane risk of bias tools and Newcastle-Ottawa Scale (NOS) were used for bias assessment depending on study design [7]. A follow-up of at least 30 days was pre-requisite for study inclusion and therefore defined as a reasonable timing for assessing clinical outcomes in the respective NOS item [7].

### Statistical analysis

Outcome distribution is displayed as binary variable across reperfusion strategies, with count and percentages. Chi-square test was used to compare prevalence of primary and secondary outcomes depending on reperfusion strategy. Odds ratio (OR) were used to provide estimate of each treatment effect on predefined outcome vs other treatment paradigms, with logistic function implemented to calculate 95% confidence intervals and *p*- alue, set as < 0.05 for significance. Heterogeneity was tested and quantified according to Q-statistics [8]. Outcome rates were meta-analyzed via random-effect modeling due to substantial heterogeneity in study design, treatment, and time-windows. Meta-regression analysis was programmed to evaluate age, NIHSS, onset-to-needle, and onset-to-reperfusion timing. Statistical analysis was performed with R-v3.3.1.

## Results

**Fig. 1:**
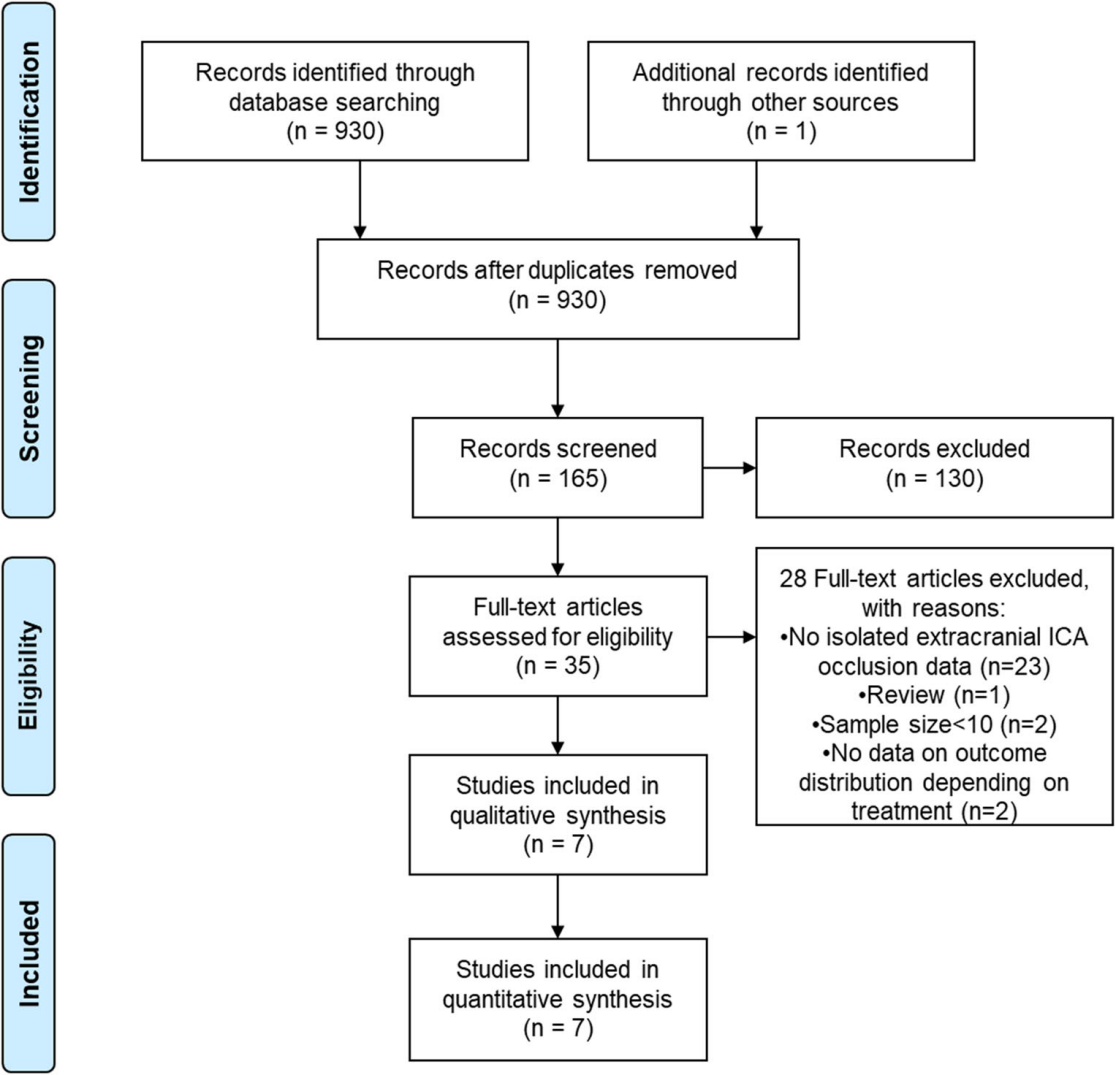
Study selection flow diagram

Overall, 165 records were screened (Fig. 1). After exclusion of reports not providing data on isolated extracranial ICA occlusion or outcome (Supplemental material - Table I), 7 studies were retrieved and included and qualitative and quantitative synthesis (Fig. 1).[2, 3, 9–13]. All studies were observational in nature, 5 of them with retrospective [9–13] and 2 with multicenter prospective design [2, 3]. None of the studies was randomized or controlled, and treatment-provided followed guidelines available at that moment as well as stroke physician decisions. Bias assessment with NOS highlighted good quality for only 2 studies [2, 3], the remaining having low to fair quality (Supplemental material – Table II). Three studies reported on IVT only [2, 11, 13], while 4 studies reported on multiple reperfusion strategies [3, 9, 10, 12].

Six studies provided data for IVT in acute ischemic stroke due to isolated extracranial ICA occlusion (*n* = 410) [2, 3, 9–11, 13]. All studies used rtPA for the treatment protocol, except for 1 study which also included patients receiving systemic urokinase [9]. Favorable outcome was defined as modified Rankin Scale (mRS) 0–2 at follow-up (ranging 1 to 3 months), except from one study which used more stringent definition (mRS 0–1) [13]. Pooling data, favorable outcome was reported in 28% (*n* = 115) after IVT, with sICH happening in 6.1% (*n* = 25), and mortality rate of 25.1% (*n* = 103). Similar proportions were confirmed by meta-analysis, with high heterogeneity for mortality (*I*^2^ = 65%, *p*_heterogeneity_ = 0.22) and low heterogeneity for functional outcome and sICH (Supplemental material – Figure I). Secondary outcome had inconsistent definition across studies, and highly variable rates of successful recanalization were reported, with pooled estimate of 14.6% (Table 1).

**Table 1:** Studies of intravenous thrombolysis (IVT) in acute ischemic stroke due to isolated cervical internal carotid artery occlusion

Study	Treatment description	Sample (*n*)	Definition of favorable outcome	Favorable outcome *n* (%)	Mortality*n* (%)	sICH*n* (%)	Definition of successful recanalization	Successful recanalization, *n* (%)
Endo 1998	IV urokinase or tPA within 6 h	12	mRS 0–2 at 1 month	0 (0%)	9 (75%)	0 (0%)	“successful” (DSA)	0 (0%)
Gliem 2017	IV tPA (no timing specified)	10	mRS 0–2 at 3 months	3 (30%)	2 (20%)	0 (0%)	“successful” (US)	0 (0%)
Paciaroni 2012	IV tPA within 4.5 h	253	mRS 0–2 at 3 months	73 (28.9%)	65 (25.7%)	12 (4.7%)	NA	NA
Paciaroni 2015	IV tPA within 4.5 h	20	mRS 0–2 at 3 months	7 (35%)	2 (10%)	1 (5%)	NA	NA
Rudolf 1999	IV tPA within 3 h	15	mRS 0–2 at 3 months	5 (33.3%)	4 (26.7%)	0 (0%)	“successful” (US)	6 (40%)
Yeo 2016	IV tPA within 4.5 h	100	mRS ≤ 1 at 3 months	27 (27%)	21 (21%)	12 (12%)	Arterial occlusive lesion grading 2–3	54 (54%)
Overall		410		115 (28%)	103 (25.1%)	25 (6.1%)		60 (14.6%)

DSA, digital subtraction angiography; *IV*, intravenous; *mRS*, modified Rankin Scale; *NA*, not available; *sICH*, symptomatic intracerebral hemorrhage; *tPA*, tissue plasminogen activator; *US*, carotid ultrasound assessment

Three studies provided data for EVT in acute ischemic stroke due to isolated cervical ICA occlusion (*n* = 150) [3, 9, 12]. EVT highly varied, including IA rtPA, thrombectomy, aspiration, stenting, and angioplasty. Data on clinical outcomes were provided by two studies only (*n* = 145), with favorable outcome (mRS 0–2) reported in 31% (*n* = 45). sICH rate was 13.8% (*n* = 20), and mortality 23.4% (*n* = 34). Similar proportions were confirmed by meta-analysis, with high heterogeneity for mortality (*I*^2^ = 86%, *p*_heterogeneity_ < 0.01) and low heterogeneity for functional outcome and sICH (Supplemental material – Figure II). Secondary outcome had inconsistent definition on the two studies reporting recanalization rate, with estimate of 46.2% (Table 2).

**Table 2:** Studies of endovascular treatment (EVT) in acute ischemic stroke due to isolated cervical internal carotid artery occlusion

Study	Treatment description	*n*	Definition of Favorable Outcome	Favorable outcome, n (%)	Mortality, n (%)	sICH, n (%)	Definition of successful recanalization	Successful recanalization, n (%)
Endo 1998	IA tPA or urokinase, angioplasty	21	mRS 0–2 at 1 month	5 (23.8%)	10 (47.6%)	1 (4.8%)	“successful”	8 (38.1%)
Paciaroni 2015	IA tPA, mechanical thrombectomy, aspiration thrombectomy, stenting	124	mRS 0–2 at 3 months	40 (32.3%)	24 (19.4%)	19 (15.3%)	NA	NA
Widimsky 2017	IA lytics, mechanical thrombectomy, carotid stent placement	5	NA	NA	NA	NA	TICI 2a or above	4 (80%)
Overall		150		45 (31%)	34 (23.4%)	20 (13.8%)		12 (46.2%)

*BI*, Barthel Index; *IA*, intraarterial; *IVT*, intravenous; *mRS*, modified Rankin scale; *sICH*, symptomatic intracerebral hemorrhage; *tPA*, tissue plasminogen activator

Three studies provided data for bridging treatment (IVT + EVT) (*n* = 62) [3, 10, 12]. Data on clinical outcomes were provided by two studies only (*n* = 56) [3, 10], with favorable outcome (mRS 0–2) reported in 46.4% (*n* = 26), sICH in 25% (*n* = 14), and mortality in 19.6% (*n* = 11). Similar proportions were confirmed by meta-analysis, with low heterogeneity (Supplemental material – Figure III). Secondary outcome was inconsistently reported, with estimate of 94.4% (Table 3).

**Table 3:** Studies of bridging treatment (intravenous thrombolysis -IVT- plus endovascular treatment -EVT-) in acute ischemic stroke due to isolated cervical internal carotid artery occlusion

Study	Treatment description	*n*	Definition of favorable outcome	Favorable outcome *n* (%)	Mortality *n* (%)	sICH *n* (%)	Definition of successful recanalization	Successful recanalization, *n* (%)
Gliem 2017	IVT + EVT	12	90 days mRS ≤ 2	6 (50%)	2 (16.7%)	3 (25%)	"successful" according to carotid ultrasound	11 (91.7%)
Paciaroni 2015	IVT + EVT	44	90 days mRS ≤ 2	20 (45.5%)	9 (20.5%)	11 (25%)	NA	NA
Widimsky 2017	IVT + EVT	6	NA	NA	NA	NA	"successful"	6 (100%)
Overall		62		26 (46.4%)	11 (19.6%)	14 (25%)		17 (94.4%)

*Legend.* IVT, intravenous; EVT, endovascular treatment; mRS, modified Rankin scale; sICH, symptomatic intracerebral hemorrhage.

Comparing pooled estimates of primary and secondary outcomes depending on reperfusion strategy (Table 4), IVT did not differ for favorable outcome, mortality, and successful recanalization compared to EVT, though having lower rate of sICH (OR 0.4, 95% CI 0.2–0.8). Compared to IVT, bridging (IVT + EVT) was associated with higher rate of favorable outcome (OR 2.2, 95% CI 1.3–3.7) and sICH (OR 5.1, 95% CI 2.5–10.5), though not impacting on mortality (OR 0.7, 95% CI 0.4–1.4). Compared to EVT, bridging (IVT + EVT) provided higher rate of favorable outcome (OR 1.9, 95% CI 1.1–3.4) and successful recanalization (OR 19.8, 95% CI 7.7–51.4), with a marginally increased risk of sICH (OR 2.1, 95% CI 1–4.4) but similar mortality rates (Table 4). Compared to IVT alone, any EVT (EVT or IVT + EVT) (*n* = 201) resulted in marginally more frequent favorable outcome (OR 1.4, 95% CI 1.0–2.0) and higher successful recanalization rates (OR 2.5, 95% CI 1.4–4.3), though increasing sICH (OR 3.1, 95% CI 1.8–5.4) (Table 4). Similar results were obtained comparing pooled estimates from random-effect meta-analysis (Supplemental material – Table III), which were partially limited by heterogeneity, sample size, and reporting biases (Supplemental material, Figures I–IV). Number of studies included (< 10) precluded meta-regression analysis.

**Table 4:** Comparison of outcomes depending on reperfusion strategy

	EVT *n* (%)	IVT *n* (%)	OR (95% CI)	*p* value
Favorable outcome	45 (31%)	115 (28%)	1.2 (0.8–1.7)	0.4953
Mortality	34 (23.4%)	103 (25.1%)	0.9 (0.6–1.4)	0.6879
sICH	20 (13.8%)	25 (6.1%)	2.5 (1.3–4.6)	0.0045
Successful recanalization	12 (46.2%)	60 (43.8%)	1.1 (0.5–2.3)	0.8244
	IVT + EVT *n* (%)	IVT *n* (%)	OR (95% CI)	*p* value
Favorable outcome	26 (46.4%)	115 (28%)	2.2 (1.3–3.7)	0.0058
Mortality	11 (19.6%)	103 (25.1%)	0.7 (0.4–1.4)	0.3725
sICH	14 (25%)	25 (6.1%)	5.1 (2.5–10.5)	< 0.0001
Successful recanalization	17 (94.4%)	60 (43.8%)	21.8 (10.5–45.2)	0.0031
	IVT + EVT *n* (%)	EVT *n* (%)	OR (95% CI)	*p* value
Favorable outcome	26 (46.4%)	45 (31%)	1.9 (1.1–3.4)	0.0421
Mortality	11 (19.6%)	34 (23.4%)	0.8 (0.4–1.7)	0.5623
sICH	14 (25%)	20 (13.8%)	2.1 (1–4.4)	0.0608
Successful recanalization	17 (94.4%)	12 (46.2%)	19.8 (7.7–51.4)	0.0067
	All EVT* *n* (%)	IVT *n* (%)	OR (95% CI)	*p* value
Favorable outcome	71 (35.3%)	115 (28%)	1.4 (1–2)	0.067
Mortality	45 (22.4%)	103 (25.1%)	0.9 (0.6–1.3)	0.459
sICH	34 (16.9%)	25 (6.1%)	3.1 (1.8–5.4)	< 0.0001
Successful recanalization	29 (65.9%)	60 (43.8%)	2.5 (1.4–4.3)	0.012

*Includes IVT + EVT and EVT without prior IVT; *CI*, confidence interval; *EVT*, endovascular treatment; *IVT*, intravenous; *OR*, odds ratio; *sICH*, symptomatic intracerebral hemorrhage

## Discussion

Our analysis suggests that a bridging approach to stroke caused by acute isolated cervical ICA occlusion might confer higher rates of favorable outcomes compared to IVT or EVT alone. However, this comes at the cost of a higher risk of sICH, though not impacting on mortality. IVT or EVT yielded similar efficacy outcomes, but EVT was related to higher odds of symptomatic intracranial bleeding.

Our results add to the available literature and refine findings from previous reports on reperfusion strategies in large vessel occlusion. Higher rates of reperfusion and improvement in functional outcome have been reported with bridging treatment compared to IVT alone [6, 14–18]. However, there is still vast debate on the role of IVT before EVT on large vessel occlusion, with a reverberating hypothesis of a potential hazard with IVT as opposed to a 10% chance of recanalization with IVT only [6, 19]. Isolated cervical ICA occlusion has been to some extent neglected by RCTs and large-scale studies, with the bulk of the literature addressing, as large vessels, distal ICA, tandem occlusion, and proximal middle cerebral artery segments [6, 19]. Therefore, the optimal management of acute stroke due to cervical ICA occlusion remains elusive. A previous systematic review on ICA occlusion suggested a possible benefit of EVT over IVT in terms of functional outcome [4]. However, all studies addressing ICA occlusion were pooled together, with consistent variations on occlusion site (cervical, terminus, intracranial) and differences in concomitant intracranial vessel occlusion (tandem), and EVT treatment data were derived merging bridging and EVT alone, therefore limiting the clinical implications of the results [4]. Our results, deriving from a systematic review with predefined protocol and stringent inclusion criteria, highlight that, when facing isolated cervical ICA occlusion, bridging with IVT + EVT might improve functional outcome. Such treatment approach is further supported by the fact that a slight increase in sICH compared to IVT does not lead to higher mortality, possibly suggesting that EVT after IVT adds to the chances of recovery without impacting survival.

However, the results of our systematic review need to be considered in the light of several limitations. First, the quality of studies included is generally low, and prevents from drawing firm conclusions on the optimal reperfusion strategy in patients with stroke due to isolated cervical ICA occlusion. Given current guidelines, supporting the use of bridging in large vessel occlusion within appropriate timing, it would be reasonable to explore if differences in functional outcome can be confirmed comparing direct EVT vs bridging treatment in an ad-hoc designed trial. Unfortunately, even latest trials (e.g., DIRECT-MT [20]) did not include patients with isolated cervical occlusion. Given an absolute difference in good functional outcome of 15.4%, we anticipate that a total sample size of 312 patients with acute ischemic stroke due to isolated cervical ICA occlusion allocated 1:1 in equal groups would yield a power of 80% to detect a significant (*p* < 0.05) improvement in favorable outcome, defined as mRS 0–2 at 3 months. As a second limitation of the study, several factors might have influenced the benefit of each treatment approach. Indeed, studies slightly differed in terms of age, NIHSS score at admission, and treatment timing. In this study, we were unable to adjust for such potential bias with meta-regression due to the small number of available papers. However, it is reasonable to hypothesize that the earlier the treatment the higher the benefit, as cervical ICA occlusion might associate with broad hemispheric penumbra. To this extent, the higher rates of functional outcomes in studies with 3–4.5 h treatment time window vs those with longer timing [9] seems to corroborate the hypothesis, although the need for RCTs on this population remains crystal-clear. Third, we did not include surgical approach, such as embolectomy or endarterectomy approach, in the hyperacute setting of isolated cervical carotid artery occlusion. However, reports on such approach are rather isolated, and need refinement through observational and randomized studies. As a fourth limitation, EVT technique highly varied across studies, and has definitely expanded in the last decade. Therefore, we might suppose that sICH could have been overestimated, given the use of IA thrombolytics, and that, with development of more effective devices, direct EVT could provide even higher rates of recanalization and lower risk of bleeding within narrow onset-to-intervention windows. Fifth, only few studies provided data on isolated cervical ICA occlusion treatment, no mRS shift analysis was available to interpolate data from different studies, and overall sample size was too restricted to allow firm generalizability of results. To this extent, it seems mandatory, in the near future as well as in the long-term, to promote full data sharing, even in the form of global repositories or supplementary material, to help limiting reporting bias. Finally, we only report unadjusted associations of EVT, IVT and bridging therapy with safety and efficacy outcomes due to lack of detailed data.

## Conclusion

Overall, our results highlight that, in acute ischemic stroke associated with isolated cervical ICA occlusion, compared to IVT or EVT alone, bridging (IVT + EVT) might lead to higher rate of functional independence at follow-up, without increasing mortality. The analysis is limited by quality and numerosity of studies, which prevented meta-regression analysis. Larger trials are critically needed to define optimal treatment for patients with stroke due to isolated cervical ICA occlusion.

## Electronic supplementary material

ESM 1 (DOCX 180 kb).

## References

[1] Powers WJ, Rabinstein AA, Ackerson T, Adeoye OM, Bambakidis NC, Becker K, Biller J, Brown M, Demaerschalk BM, Hoh B, Jauch EC, Kidwell CS, Leslie-Mazwi TM, Ovbiagele B, Scott PA, Sheth KN, Southerland AM, Summers DV, Tirschwell DL (2018). Guidelines for the early management of patients with acute ischemic stroke: a guideline for healthcare professionals from the American Heart Association/American Stroke Association. Stroke.

[2] Paciaroni M, Balucani C, Agnelli G, Caso V, Silvestrelli G, Grotta JC, Demchuk AM, Sohn SI, Orlandi G, Leys D, Pezzini A, Alexandrov AV, Silvestrini M, Fofi L, Barlinn K, Inzitari D, Ferrarese C, Tassi R, Tsivgoulis G, Consoli D, Baldi A, Bovi P, Luda E, Galletti G, Invernizzi P, DeLodovici ML, Corea F, del Sette M, Monaco S, Marcheselli S, Alberti A, Venti M, Acciarresi M, D'Amore C, Macellari F, Lanari A, Previdi P, Gonzales NR, Pandurengan RK, Vahidy FS, Sline M, Bal SS, Chiti A, Gialdini G, Dumont F, Cordonnier C, Debette S, Padovani A, Cerqua R, Bodechtel U, Kepplinger J, Nesi M, Nencini P, Beretta S, Trentini C, Martini G, Piperidou C, Heliopoulos I, D'Anna S, Cappellari M, Donati E, Bono G, Traverso E, Toni D (2012). Systemic thrombolysis in patients with acute ischemic stroke and internal carotid artery occlusion: the ICARO study. Stroke.

[3] Paciaroni M, Inzitari D, Agnelli G, Caso V, Balucani C, Grotta JC, Sarraj A, Sung-Il S, Chamorro A, Urra X, Leys D, Henon H, Cordonnier C, Dequatre N, Aguettaz P, Alberti A, Venti M, Acciarresi M, D’Amore C, Zini A, Vallone S, Dell’Acqua ML, Menetti F, Nencini P, Mangiafico S, Barlinn K, Kepplinger J, Bodechtel U, Gerber J, Bovi P, Cappellari M, Linfante I, Dabus G, Marcheselli S, Pezzini A, Padovani A, Alexandrov AV, Shahripour RB, Sessa M, Giacalone G, Silvestrelli G, Lanari A, Ciccone A, de Vito A, Azzini C, Saletti A, Fainardi E, Orlandi G, Chiti A, Gialdini G, Silvestrini M, Ferrarese C, Beretta S, Tassi R, Martini G, Tsivgoulis G, Vasdekis SN, Consoli D, Baldi A, D’Anna S, Luda E, Varbella F, Galletti G, Invernizzi P, Donati E, de Lodovici ML, Bono G, Corea F, Sette MD, Monaco S, Riva M, Tassinari T, Scoditti U, Toni D (2015). Intravenous thrombolysis or endovascular therapy for acute ischemic stroke associated with cervical internal carotid artery occlusion: the ICARO-3 study. J Neurol.

[4] Mokin M, Kass-Hout T, Kass-Hout O, Dumont TM, Kan P, Snyder KV, Hopkins LN, Siddiqui AH, Levy EI (2012). Intravenous thrombolysis and endovascular therapy for acute ischemic stroke with internal carotid artery occlusion: A systematic review of clinical outcomes. Stroke.

[5] Linfante I, Llinas RH, Selim M, Chaves C, Kumar S, Parker RA, Caplan LR, Schlaug G (2002). Clinical and vascular outcome in internal carotid artery versus middle cerebral artery occlusions after intravenous tissue plasminogen activator. Stroke.

[6] Tsivgoulis G, Katsanos AH, Schellinger PD, Köhrmann M, Varelas P, Magoufis G, Paciaroni M, Caso V, Alexandrov AW, Gurol E, Alexandrov AV (2018). Successful reperfusion with intravenous thrombolysis preceding mechanical thrombectomy in large-vessel occlusions. Stroke.

[7] Luchini C, Stubbs B, Solmi M (2017). Assessing the quality of studies in meta-analyses: advantages and limitations of the Newcastle Ottawa Scale. World J Metaanal.

[8] Higgins JPT, Thompson SG (2002). Quantifying heterogeneity in a meta-analysis. Stat Med.

[9] Endo S, Kuwayama N, Hirashima Y, Akai T, Nishijima M, Takaku A (1998). Results of urgent thrombolysis in patients with major stroke and atherothrombotic occlusion of the cervical internal carotid artery. Am J Neuroradiol.

[10] Gliem M, Lee JI, Barckhan A, Turowski B, Hartung HP, Jander S (2017). Outcome and treatment effects in stroke associated with acute cervical ICA occlusion. PLoS One.

[11] Rudolf J, Neveling M, Grond M, Schmulling S, Stenzel C, Heiss WD (1999). Stroke following internal carotid artery occlusion - A contra-indication for intravenous thrombolysis?. Eur J Neurol.

[12] Widimsky P, Koznar B, Peisker T, Vasko P, Rohac F, Vavrova J, Kroupa J, Stetkarova I (2017). Feasibility and safety of direct catheter-based thrombectomy in the treatment of acute ischaemic stroke. Cooperation among cardiologists, neurologists and radiologists. Prospective registry Prague-16. EuroIntervention.

[13] Yeo LLL, Kong WY, Paliwal P, Teoh HL, Seet RC, Soon D, Rathakrishnan R, Ong V, Lee TH, Wong HF, Chan BPL, Leow WK, Yuan C, Ting E, Gopinathan A, Tan BYQ, Sharma VK (2016). Intravenous thrombolysis for acute ischemic stroke due to cervical internal carotid artery occlusion. J Stroke Cerebrovasc Dis.

[14] Katsanos AH, Malhotra K, Goyal N, Arthur A, Schellinger PD, Köhrmann M, Krogias C, Turc G, Magoufis G, Leys D, Ahmed N, Khatri P, Goyal M, Alexandrov AV, Tsivgoulis G (2019). Intravenous thrombolysis prior to mechanical thrombectomy in large vessel occlusions. Ann Neurol.

[15] Katsanos AH, Tsivgoulis G (2019). Is intravenous thrombolysis still necessary in patients who undergo mechanical thrombectomy?. Curr Opin Neurol.

[16] Goyal N, Tsivgoulis G, Frei D, Turk A, Baxter B, Froehler MT, Mocco J, Pandhi A, Zand R, Malhotra K, Hoit D, Elijovich L, Loy D, Turner RD, Mascitelli J, Espaillat K, Katsanos AH, Alexandrov AW, Alexandrov AV, Arthur AS (2018). Comparative safety and efficacy of combined ivt and mt with direct mt in large vessel occlusion. Neurology.

[17] Wollenweber FA, Tiedt S, Alegiani A, Alber B, Bangard C, Berrouschot J, Bode FJ, Boeckh-Behrens T, Bohner G, Bormann A, Braun M, Dorn F, Eckert B, Flottmann F, Hamann GF, Henn KH, Herzberg M, Kastrup A, Kellert L, Kraemer C, Krause L, Lehm M, Liman J, Lowens S, Mpotsaris A, Papanagiotou P, Petersen M, Petzold GC, Pfeilschifter W, Psychogios MN, Reich A, von Rennenberg R, Röther J, Schäfer JH, Siebert E, Siedow A, Solymosi L, Thonke S, Wagner M, Wunderlich S, Zweynert S, Nolte CH, Gerloff C, Thomalla G, Dichgans M, Fiehler J (2019). Functional outcome following stroke thrombectomy in clinical practice. Stroke.

[18] Chalos V, LeCouffe NE, Uyttenboogaart M (2019). Endovascular treatment with or without prior intravenous alteplase for acute ischemic stroke. J Am Heart Assoc.

[19] Vidale S, Romoli M, Consoli D, Agostoni EC (2020) Bridging versus Direct Mechanical Thrombectomy in Acute Ischemic Stroke: A Subgroup Pooled Meta-Analysis for Time of Intervention, Eligibility, and Study Design. Cerebrovasc Dis 24:1–10 10.1159/000507844 32335550

[20] Yang P, Zhang Y, Zhang L, Zhang Y, Treurniet KM, Chen W, Peng Y, Han H, Wang J, Wang S, Yin C, Liu S, Wang P, Fang Q, Shi H, Yang J, Wen C, Li C, Jiang C, Sun J, Yue X, Lou M, Zhang M, Shu H, Sun D, Liang H, Li T, Guo F, Ke K, Yuan H, Wang G, Yang W, Shi H, Li T, Li Z, Xing P, Zhang P, Zhou Y, Wang H, Xu Y, Huang Q, Wu T, Zhao R, Li Q, Fang Y, Wang L, Lu J, Li Y, Fu J, Zhong X, Wang Y, Wang L, Goyal M, Dippel DWJ, Hong B, Deng B, Roos YBWEM, Majoie CBLM, Liu J (2020). Endovascular thrombectomy with or without intravenous alteplase in acute stroke. N Engl J Med.

