# Supplementary material for: Reperfusion strategies in stroke due to isolated cervical internal carotid artery occlusion: systematic review and treatment comparison
Source: Neurol Sci. 2020 Oct 10;42(6):2301–8. doi: 10.1007/s10072-020-04735-5 (PMC8159826; doi:10.1007/s10072-020-04735-5)
Supplement: Supplementary file 1 — (DOCX 180 kb). [file 10072_2020_4735_MOESM1_ESM.docx]

**SUPPLEMENTAL MATERIAL**

**Supplemental material – Table I. Excluded studies and reasons for exclusion.**

| **Study** | **Treatment Description** | **Sample** (n) | **Reason for exclusion** |
| --- | --- | --- | --- |
| Arnold 2003^1^ | IA urokinase, angioplasty, stenting | 24 | no isolated extracranial ICA occlusion data |
| Arnold 2007^2^ | IA urokinase | 43 | no isolated extracranial ICA occlusion data |
| Costalat 2011^3^ | Mechanical thrombectomy | 14 | no isolated extracranial ICA occlusion data |
| Dabitz 2007^4^ | IA tPA, angioplasty, stenting | 10 | no isolated extracranial ICA occlusion data |
| Ernst 2000^5^ | IA tPA | 6 | sample size<10 |
| Fesl 2011^6^ | IA tPA, mechanical thrombectomy, aspiration thrombectomy, stenting | 14 | no isolated extracranial ICA occlusion data |
| Hauck 2011^7^ | IA tPA, mechanical thrombectomy, stenting | 22 | no distribution of outcome depending on treatment |
| Hong 2014^8^ | IVT, IVT+EVT, EVT | 70/66/35 | no isolated extracranial ICA occlusion data |
| Imai 2006^9^ | IA urokinase, angioplasty, mechanical thrombectomy, aspiration thrombectomy | 3 | sample size<10 |
| Jovin 2005^10^ | Angioplasty, stenting | 15 | no distribution of outcome depending on treatment |
| Kim 2008^11^ | IA urokinase | 10 | no isolated extracranial ICA occlusion data |
| Kimura 2009^12^ | IV tPA within 3 h | 21 | no isolated extracranial ICA occlusion data |
| Kwak JH et al, 2014^13^ | IVT+EVT | 22 | no isolated extracranial ICA occlusion data |
| Lin 2009^14^ | IA tPA or urokinase, angioplasty, stenting, mechanical thrombectomy | 75 | no isolated extracranial ICA occlusion data |
| Malik 2011^15^ | IA tPA or urokinase, angioplasty, stenting, mechanical thrombectomy, aspiration thrombectomy | 77 | no isolated extracranial ICA occlusion data |
| Miteff 2011^16^ | IA urokinase, angioplasty, stenting, mechanical thrombectomy | 11 | no isolated extracranial ICA occlusion data |
| Mokin 2012^17^ | IVT, EVT | review | review |
| Nedeltchev 2005^18^ | IA urokinase, aspiration thrombectomy, stenting | 25 | no isolated extracranial ICA occlusion data |
| Rubiera 2006^19^ | IV tPA within 3 h | 44 | no isolated extracranial ICA occlusion data |
| Saqqur 2007^20^ | IV tPA within 3 h | 14 | no isolated extracranial ICA occlusion data |
| Shi 2010^21^ | IA tPA, mechanical thrombectomy | 99 | no isolated extracranial ICA occlusion data |
| Sorimachi 2004^22^ | IA urokinase, mechanical thrombectomy | 11 | no isolated extracranial ICA occlusion data |
| Suh 2007^23^ | IA fibrinolytic thrombolysis, angioplasty, stenting, mechanical thrombectomy | 33 | no isolated extracranial ICA occlusion data |
| Urbach 1997^24^ | IA urokinase | 12 | no isolated extracranial ICA occlusion data |
| Watanabe 2011^25^ | IA urokinase, angioplasty | 10 | no isolated extracranial ICA occlusion data |
| Wunderlich 2005^26^ | IV tPA within 3 h (n = 12) and 6 h (n = 3) | 15 | no isolated extracranial ICA occlusion data |
| Yoo 2009^27^ | IA urokinase, angioplasty, stenting, mechanical thrombectomy | 16 | no isolated extracranial ICA occlusion data |
| Zaidat 2002^28^ | IA tPA or urokinase, mechanical thrombectomy | 18 | no isolated extracranial ICA occlusion data |

**Supplemental material – Table II. Risk of bias in included studies.**

| **Study** | **Design** | **Reperfusion strategies explored** | **Selection** | | | | **Comparability** | **Outcome** | | | **Overall score** | **Overall quality** |
| --- | --- | --- | --- | --- | --- | --- | --- | --- | --- | --- | --- | --- |
|  |  |  | Representativeness of the exposed cohort | Selection of the non-exposed/comparator cohort | Ascertainment of exposure | Demonstration outcome-free at baseline |  | Assessment of outcome | Was follow-up long enough for outcomes to occur | Adequacy of follow up of cohorts |  |  |
| Endo 1998^29^ | retrospective, single center | IVT, EVT | 0 | 1 | 1 | 0 | 0 | 1 | 1 | 1 | 5 | low |
| Gliem 2017^30^ | retrospective, single center | IVT, EVT | 1 | 1 | 1 | 1 | 1 | 1 | 1 | 1 | 8 | good |
| Paciaroni 2012^31^ | prospective, multicentre | IVT | 1 | 0 | 1 | 1 | 1 | 1 | 1 | 1 | 7 | fair |
| Paciaroni 2015^32^ | prospective, multicentre | IVT, EVT, IVT+EVT | 1 | 1 | 1 | 1 | 2 | 1 | 1 | 1 | 9 | good |
| Rudolf 1999^33^ | retrospective, single center | IVT | 0 | 0 | 1 | 0 | 0 | 1 | 1 | 1 | 4 | low |
| Widimsky 2017^34^ | retrospective, single center | IVT+EVT, EVT | 0 | 1 | 1 | 0 | 0 | 1 | 1 | 1 | 5 | low |
| Yeo 2016^35^ | retrospective, single center | IVT | 1 | 1 | 1 | 0 | 0 | 1 | 1 | 1 | 6 | fair |

**Supplemental material – Figure I. Forest plot of proportions of favorable outcome (top), mortality (center) and symptomatic intracerebral hemorrhage (sICH) (bottom) after intravenous thrombolysis.**

Legend. IVT, intravenous thrombolysis; sICH, symptomatic intracerebral hemorrhage.

**Supplemental material – Figure II. Forest plot of proportions of favorable outcome (top), mortality (center) and symptomatic intracerebral hemorrhage (sICH) (bottom) after endovascular treatment.**

Legend. EVT, endovascular treatment; sICH, symptomatic intracerebral hemorrhage.

**Supplemental material – Figure III. Forest plot of proportions of favorable outcome (top), mortality (center) and symptomatic intracerebral hemorrhage (sICH) (bottom) after bridging.**

Legend. EVT, endovascular treatment; IVT, intravenous thrombolysis; sICH, symptomatic intracerebral hemorrhage.

**Supplemental material – Figure IV. Funnel plots for included studies depending on reperfusion strategy (IVT left, EVT middle, IVT+EVT right)**

**Supplementary material – Table III. Favorable outcome depending on reperfusion strategy, comparing data from pooled random-effect meta-analysis of proportion.**

|  | **EVT** | **IVT** | **OR (95%CI)** | **p-value** |
| --- | --- | --- | --- | --- |
|  | **estimate,**% **(95%CI)** | **estimate,**% **(95%CI)** |  |  |
| **favorable outcome from meta-analysis pooled estimates** | 31.1 (24.1-39.1) | 28.7 (24.4-33-3) | 1.1 (0.8-1.7) | 0.5 |
|  |  |  |  |  |
|  | **IVT+EVT** | **IVT** | **OR (95%CI)** | **p-value** |
|  | **estimate,**% **(95%CI)** | **estimate,**% **(95%CI)** |  |  |
| **favorable outcome from meta-analysis pooled estimates** | 46.4 (33.9-59.5) | 28.7 (24.4-33-3) | 2.2 (1.3-3.6) | **0.006** |
|  |  |  |  |  |
|  | **IVT+EVT** | **EVT** | **OR (95%CI)** | **p-value** |
|  | **estimate,**% **(95%CI)** | **estimate,**% **(95%CI)** |  |  |
| **favorable outcome from meta-analysis pooled estimates** | 46.4 (33.9-59.5) | 31.1 (24.1-39.1) | 1.9 (1.1-3.4) | **0.04** |

**References**

1. Arnold M, Nedeltchev K, Mattle HP. [Anticoagulation and antiaggregation in neurological patients]. *Ther. Umsch.* 2003;60:33–35.

2. Arnold M, Kappeler L, Nedeltchev K, Brekenfeld C, Fischer U, Keserue B, et al. Recanalization and outcome after intra-arterial thrombolysis in middle cerebral artery and internal carotid artery occlusion: Does sex matter? *Stroke*. 2007;38:1281–1285.

3. Costalat V, MacHi P, Lobotesis K, Maldonado I, Vendrell JF, Riquelme C, et al. Rescue, combined, and stand-alone thrombectomy in the management of large vessel occlusion stroke using the solitaire device: A prospective 50-patient single-center study: Timing, safety, and efficacy. *Stroke*. 2011;42:1929–1935.

4. Dabitz R, Triebe S, Leppmeier U, Ochs G, Vorwerk D. Percutaneous recanalization of acute internal carotid artery occlusions in patients with severe stroke. *Cardiovasc. Intervent. Radiol.* 2007;30:34–41.

5. Ernst R, Pancioli A, Tomsick T, Kissela B, Woo D, Kanter D, et al. Combined intravenous and intra-arterial recombinant tissue plasminogen activator in acute ischemic stroke. *Stroke*. 2000;31:2552–2557.

6. Fesl G, Wiesmann M, Patzig M, Holtmannspoetter M, Pfefferkorn T, Dichgans M, et al. Endovascular mechanical recanalisation of acute carotid-t occlusions: A single-center retrospective analysis. *Cardiovasc. Intervent. Radiol.* 2011;34:280–286.

7. Hauck EF, Natarajan SK, Ohta H, Ogilvy CS, Hopkins LN, Siddiqui AH, et al. Emergent endovascular recanalization for cervical internal carotid artery occlusion in patients presenting with acute stroke. *Neurosurgery*. 2011;69:899–907.

8. Hong JH, Kang J, Jang MU, Kim BJ, Han MK, Park TH, et al. Recanalization therapy for internal carotid artery occlusion presenting as acute ischemic stroke. *J. Stroke Cerebrovasc. Dis.* 2014;23:2183–2189.

9. Imai K, Mori T, Izumoto H, Takabatake N, Kunieda T, Shimizu H, et al. Clot removal therapy by aspiration and extraction for acute embolic carotid occlusion. *Am. J. Neuroradiol.* 2006;27:1521–1527.

10. Jovin TG, Gupta R, Uchino K, Jungreis CA, Wechsler LR, Hammer MD, et al. Emergent stenting of extracranial internal carotid artery occlusion in acute stroke has a high revascularization rate. *Stroke*. 2005;36:2426–2430.

11. Kim DJ, Kim DI, Byun JS, Jung JY, Suh SH, Kim EY, et al. Intra-arterial thrombolytic therapy for hyperacute ischemic stroke caused by tandem occlusion. *Cerebrovasc. Dis.* 2008;26:184–189.

12. Kimura K, Iguchi Y, Shibazaki K, Iwanaga T, Aoki J. Recanalization of the MCA should play an important role in dramatic recovery after t-PA therapy in patients with ICA occlusion. *J. Neurol. Sci.* 2009;285:130–133.

13. Kwak JH, Zhao L, Kim JK, Park S, Lee D -g., Shim JH, et al. The Outcome and Efficacy of Recanalization in Patients with Acute Internal Carotid Artery Occlusion. *Am. J. Neuroradiol.* 2014;35:747–753.

14. Lin R, Vora N, Zaidi S, Aleu A, Jankowitz B, Thomas A, et al. Mechanical approaches combined with intra-arterial pharmacological therapy are associated with higher recanalization Rates Than Either Intervention Alone in Revascularization of Acute Carotid Terminus Occlusion. *Stroke*. 2009;40:2092–2097.

15. Malik AM, Vora NA, Lin R, Zaidi SF, Aleu A, Jankowitz BT, et al. Endovascular treatment of tandem extracranial/intracranial anterior circulation occlusions: Preliminary single-center experience. *Stroke*. 2011;42:1653–1657.

16. Miteff F, Faulder KC, Goh ACC, Steinfort BS, Sue C, Harrington TJ. Mechanical thrombectomy with a self-expanding retrievable intracranial stent (solitaire ab): Experience in 26 patients with acute cerebral artery occlusion. *Am. J. Neuroradiol.* 2011;32:1078–1081.

17. Mokin M, Kass-Hout T, Kass-Hout O, Dumont TM, Kan P, Snyder K V., et al. Intravenous thrombolysis and endovascular therapy for acute ischemic stroke with internal carotid artery occlusion: A systematic review of clinical outcomes. *Stroke*. 2012;43:2362–2368.

18. Nedeltchev K, Brekenfeld C, Remonda L, Ozdoba C, Do D Do, Arnold M, et al. Internal carotid artery stent implantation in 25 patients with acute stroke: Preliminary results. *Radiology*. 2005;237:1029–1037.

19. Rubiera M, Ribo M, Delgado-Mederos R, Santamarina E, Delgado P, Montaner J, et al. Tandem internal carotid artery/middle cerebral artery occlusion: An independent predictor of poor outcome after systemic thrombolysis. *Stroke*. 2006;37:2301–2305.

20. Saqqur M, Uchino K, Demchuk AM, Molina CA, Garami Z, Calleja S, et al. Site of arterial occlusion identified by transcranial Doppler predicts the response to intravenous thrombolysis for stroke. *Stroke*. 2007;38:948–954.

21. Shi ZS, Loh Y, Walker G, Duckwiler GR. Endovascular thrombectomy for acute ischemic stroke in failed intravenous tissue plasminogen activator versus non-intravenous tissue plasminogen activator patients: Revascularization and outcomes stratified by the site of arterial occlusions. *Stroke*. 2010;41:1185–1192.

22. Sorimachi T, Fujii Y, Tsuchiya N, Nashimoto T, Harada A, Ito Y, et al. Recanalization by mechanical embolus disruption during intra-arterial thrombolysis in the carotid territory. *Am. J. Neuroradiol.* 2004;25:1391–1402.

23. Suh DC, Kim JK, Choi CG, Kim SJ, Pyun HW, Ahn C, et al. Prognostic factors for neurologic outcome after endovascular revascularization of acute symptomatic occlusion of the internal carotid artery. *Am. J. Neuroradiol.* 2007;28:1167–1171.

24. Urbach H, Ries F, Ostertun B, Solymosi L. Local intra-arterial fibrinolysis in thromboembolic “T” occlusions of the internal carotid artery. *Neuroradiology*. 1997;39:105–110.

25. Watanabe M, Mori T, Imai K, Izumoto H. Endovascular interventions for patients with serious symptoms caused by embolic carotid T occlusion. *Neurol. Med. Chir. (Tokyo).* 2011;51:282–288.

26. Wunderlich MT, Stolz E, Seidel G, Postert T, Gahn G, Sliwka U, et al. Conservative medical treatment and intravenous thrombolysis in acute stroke from carotid T occlusion. *Cerebrovasc. Dis.* 2005;20:355–361.

27. Yoo AJ, Verduzco LA, Schaefer PW, Hirsch JA, Rabinov JD, González RG. MRI-based selection for intra-arterial stroke therapy: Value of pretreatment diffusion-weighted imaging lesion volume in selecting patients with acute stroke who will benefit from early recanalization. *Stroke*. 2009;40:2046–2054.

28. Zaidat OO, Suarez JI, Santillan C, Sunshine JL, Tarr RW, Paras VH, et al. Response to intra-arterial and combined intravenous and intra-arterial thrombolytic therapy in patients with distal internal carotid artery occlusion. *Stroke*. 2002;33:1821–1826.

29. Endo S, Kuwayama N, Hirashima Y, Akai T, Nishijima M, Takaku A. Results of urgent thrombolysis in patients with major stroke and atherothrombotic occlusion of the cervical internal carotid artery. *Am. J. Neuroradiol.* 1998;19:1169–1175.

30. Gliem M, Lee JI, Barckhan A, Turowski B, Hartung HP, Jander S. Outcome and treatment effects in stroke associated with acute cervical ICA occlusion. *PLoS One*. 2017;12:1–8.

31. Paciaroni M, Balucani C, Agnelli G, Caso V, Silvestrelli G, Grotta JC, et al. Systemic thrombolysis in patients with acute ischemic stroke and internal carotid artery occlusion: The ICARO study. *Stroke*. 2012;43:125–130.

32. Paciaroni M, Inzitari D, Agnelli G, Caso V, Balucani C, Grotta JC, et al. Intravenous thrombolysis or endovascular therapy for acute ischemic stroke associated with cervical internal carotid artery occlusion: the ICARO-3 study. *J. Neurol.* 2015;262:459–468.

33. Rudolf J, Neveling M, Grond M, Schmülling S, Stenzel C, Heiss WD. Stroke following internal carotid artery occlusion - A contra-indication for intravenous thrombolysis? *Eur. J. Neurol.* 1999;6:51–55.

34. Widimsky P, Koznar B, Peisker T, Vasko P, Rohac F, Vavrova J, et al. Feasibility and safety of direct catheter-based thrombectomy in the treatment of acute ischaemic stroke. Cooperation among cardiologists, neurologists and radiologists. Prospective registry Prague-16. *EuroIntervention*. 2017;13:131–136.

35. Yeo LLL, Kong WY, Paliwal P, Teoh HL, Seet RC, Soon D, et al. Intravenous Thrombolysis for Acute Ischemic Stroke due to Cervical Internal Carotid Artery Occlusion. *J. Stroke Cerebrovasc. Dis.* 2016;25:2423–2429.
